# The School as an Arena for Co-Creating Participation, Equity, and Well-Being—A Photovoice Study from Norway

**DOI:** 10.3390/ijerph18168252

**Published:** 2021-08-04

**Authors:** Camilla Ihlebæk, Camilla Castellan, Jenny Flobak, Jo Ese

**Affiliations:** 1 Department of Public Health Science, Norwegian University of Life Sciences, NMBU, 1432 Ås, Norway; camilla.castellan93@gmail.com (C.C.); jenny@flobak.net (J.F.); 2 Department of Welfare, Management and Organisation, Østfold University College, 1757 Halden, Norway; jo.ese@hiof.no

**Keywords:** community school, well-being, photovoice, co-creation, health equity, neighborhood

## Abstract

Schools may play an essential role as an arena for co-creating community activities that enhance well-being, equity, and citizenship. Still, there is limited knowledge about physical and non-physical factors that contribute to well-being within such approaches. The aim of this study was to identify important factors for well-being as perceived by pupils, school employees, and parents in a community school in Norway. The participatory method photovoice was used, and seven pupils, six employees, and four parents participated by taking photos used as the basis for six focus group discussions. Transcripts of the discussions were analyzed using Systematic Text Condensation. The analysis showed that the participants experienced that the school’s built and natural environment, the activities happening there, and the human resources and organization at the school facilitated perceptions of safety, inclusion, and cohesion, which in turn contributed to well-being. Furthermore, the results showed that co-creating schools as a community arena could be an innovative way of ensuring participation, equity, and well-being in the community. Such an approach might be especially important in deprived areas or in multi-ethnic communities. An important prerequisite to succeed is the openness of the school’s staff to engage in co-creation with other stakeholders in the community.

## 1. Introduction

It is widely recognized that health and well-being are primarily created and maintained within arenas outside the health care sector, and communities and neighborhoods are emphasized as the key settings for health promotion [1,2]. Schools are usually placed centrally within communities and have the potential for being an important meeting place for all children, parents, and residents in the neighborhood. Furthermore, as almost all children in a community will attend elementary school, schools are seen as a key arena with unique opportunities for promoting health and well-being, as well as ensuring inclusion, equity, and democratic values [3,4,5]. Schools may therefore play an essential role in solving some of society’s most complex challenges, often termed wicked problems [6]. Therefore, it is important to gain more knowledge on how elementary schools can be developed to target such problems and to gain more insight into which factors are perceived as important for creating well-being for different stakeholders using the school as a community arena.

The purpose of this study was to investigate a new framework developed in Østfold, Norway using schools as an innovative arena for co-creating community development and inclusion. Several approaches and conceptual frameworks for integrating health promotion in schools have been developed, such as Health Promoting Schools (HPS) [7], The Whole School Approach (WSA) [3], and The Whole School, Whole Community, Whole Child model (WSCC) [8]. Common for all these frameworks is an emphasis on health-promoting activities at school, such as health education, healthy nutrition, and physical activity. Furthermore, they aim at enhancing students social, emotional, cognitive, and academic skills and focus on collaborative initiatives involving and engaging teachers, parents and guardians, and the health care sector and other community resources [7,8]. Several studies have reported that approaches such as HPS, WSA, and WSCC could improve student’s health and well-being; school connectedness; academic achievements; or adverse behavior, such as bulling [9,10,11,12]. However, over time, these approaches have switched focus, not only aiming at influencing the knowledge, attitudes, behaviors, and well-being of individual students but toward more community engagement and an ecological settings approach, which focus on equity, participation, and creating empowerment processes—both within and outside of schools [7,13].

In the US in particular, the concept of community schools developed as a part of WSCC, with a focus on using schools to promote democracy and equity [14]. An important factor within community schools is the use of external partnerships to develop schools into “neighborhood hubs” [15]. Community schools could be important assets for building social capital within a community, which can enhance people’s capacities to facilitate changes in their communities [16]. In addition, high levels of social capital are found to be associated with good health, well-being, and satisfaction with life [17,18,19] and has been suggested to mediate the association between social inequality and health [20].

Community schools have similarities to other community approaches, such as the Asset-Based Community Development (ABCD) approach, which is based on an assumption that all persons have abilities and capacities and that well-being and belonging are dependent on being able to use those capabilities, which will, in turn, empower the whole community [21]. The common goal of these approaches is to enable local communities to build themselves bottom-up by utilizing local resources and capacities and building bridging and linking social capital to connect these resources [22]. The concept of community schools is therefore built upon a strong belief that public value is best created in the intercept between multiple stakeholders and by applying participatory and cross-sectional approaches, a way of organizing the public sector that in the recent literature has been popularized as co-creation [23,24,25]. Co-creation can be described as “a constructive exchange of different kinds of knowledge, re-sources, competences, and ideas” when different stakeholders in a community try to solve a shared challenge or task [26]. It has been argued that co-creation and health promotion have mutual benefits, as they emphasize collaboration, participation, context sensitivity, and knowledge-based practice, all needed to improve health and well-being [27]. Furthermore, co-creation could be especially promising for solving challenges at the local level [26].

In Norway, a substantial number of schools are certified within the HPS system, focusing on creating health-promoting activities for the pupils. However, in Østfold, a region that is part of Viken county in the southeast of Norway, a conceptual framework more similar to community schools has been developed by using schools as an innovative arena for co-creating community development and inclusion [28]. Although Norway is known as a wealthy country, there is a significant and increasing social gradient in health [29,30], and, in Østfold, there is a higher proportion of inhabitants with low socioeconomical status compared with the rest of the country [31]. As a coordinated attempt to counter inequity and foster inclusion, several schools are shifting their approach with the intention of becoming an asset for the community by opening school premises for activities with involvement and participation of the whole community and the aim to promote well-being, equity, and inclusion among the whole community. This is in accordance with recent national recommendations to reduce social inequalities in health. These recommendations emphasize the necessity of implementing universal policies accessible to all in the local communities where people live [4,32].

Although there is substantial research in the literature on HPS, WSA, WSCC, and community schools, there is still limited knowledge about which physical and non-physical factors contribute to well-being within such approaches. There is also a need for qualitative studies that can provide in-depth knowledge on how community schools influence the well-being of different stakeholders, such as teachers, pupils, and parents [14]. As community schools are a relatively new approach in Norway, no study has been conducted to describe and investigate experiences. Furthermore, although the concept of co-creation has gained traction in the research literature, as well as in the public sector in recent years, documented examples of how co-creation is performed in practice are limited [26]. The aim of this study therefore was to identify the factors in and around the school premises and organization that were perceived as important to promote well-being by pupils, school employees, and parents in a community school in Østfold, Norway and to discuss how co-creation contributed to equity and participation.

## 2. Materials and Methods

The setting was Alvimhaugen Primary School, in Sarpsborg, Norway. The school is located in an area with socioeconomic challenges related to living conditions and with a high proportion of immigrants. About 70% of the pupils have immigrant backgrounds and 19 different languages are spoken. For several years, the school has opened its premises for community activities both before and after teaching hours. All activities are free of charge and include breakfast; the leisure club “The mirror of the world” with hot meals; several different sport activities, including a physical activity group for women only; a guitar course; and a homework café [33]. The school regularly hosts special events for the whole community, such as an international café and national day celebrations. The school premises are also used for birthdays parties and playing sports, and the playground is available after school, in the evenings, and on weekends. Winter sport equipment is also available to rent free of charge [33].

The participatory qualitative method photovoice was used to collect the data [34]. This method challenges traditional relationships between the researchers and the participants, as several measures are taken to give participants the possibility to identify and define the relevant topics to discuss [35]. The method was chosen as it gives the participants a unique possibility to document and portray their everyday life and surroundings from their own viewpoint [34]. First, the participants were asked to take pictures that illustrated what they felt contributed to well-being at the school and its surroundings, and these pictures were then used to facilitate dialogue, discussion, and analysis in focus groups. The photovoice method generates a richer data material than traditional focus groups, as it includes both pictures and text material as data [36].

The data were collected between November 2017 and February 2018. A purposive sample process was applied to recruit the participants to ensure a multi-perspective approach of different stakeholders [37]. In collaboration with the school’s administration, an invitation to participate was distributed to the school’s pupil council, the staff, and the parent’s council. This resulted in a convenience sample of a total of 16 persons wanting to participate: 6 pupils (4th to 7th grade, ages 9 to 12), 6 teachers/school employees, and 4 parents. Two preparatory meetings were held to describe the project purpose and to give the instructions to take 3 to 5 pictures that illustrated what led them to experience well-being at the school and in the school premises. Participants were encouraged to base their choice of pictures on their own sense of what was significant or what they believed well-being meant. The adult participants were instructed to use their mobile phones to take the pictures, and the pupils were provided with disposable cameras. The pictures were taken over a period of two weeks and then sent to the authors.

The pictures were then used as a foundation in two subsequent focus group meetings with each of the three stakeholder groups. In the first focus group meeting, the participants jointly explored the content of the pictures. Each participant explained the motives for taking each picture and described their thoughts related to it. To ensure a rich description of the picture, a modified three question version of the tool SHOWeD was used to guide the description: What do you see here? What is happening? How does this relate to your well-being? [34,38]. These questions were then followed with a free discussion related to all of the pictures, and all participants could reflect upon each other’s pictures. In the second focus group meeting, the participants were given the task of thematizing the pictures and asked to assign the pictures to different themes, contributing to an early stage of the analysis in the project. The pupils generated 6 themes from their pictures: *friends, leisure activities, break time, safety, working peace,* and *good food*. The school employees also generated 6 themes: *happy children and adults, safety, good colleagues, humor, a good framework for teaching,* and *an including school and workplace*. The parents identified 3 themes from their pictures: *availability, cohesion,* and *safety.*

All focus group meetings were recorded and transcribed verbatim. The text data were analyzed using Systematic Text Condensation [39], a modified version of Giorgi’s phenomenological approach (Giorgi 1985). All authors participated in the analysis and interpretation of the data. Four steps were followed: transcripts were read by all authors to gain a contextualized impression of the data, and preliminary themes were identified; units of meaning from the text were coded into themes; the meaning in the coded themes was condensed; and descriptions were then summarized to establish the concepts reflecting the dimensions related to well-being. The qualitative findings are presented as descriptive summaries and illustrated by quotes from the transcripts. The quotes were translated from Norwegian and coded by group.

In addition to the text analysis, the pictures taken by the participants were analyzed during the focus groups and in the text analysis. As the pictures were used as a basis for the group discussion, the pictures and text material were closely connected. The ‘interpretive engagement’ framework was used to interpret the pictures [40]. This framework consists of three stages of visual meaning-making: through participant engagement (Stage 1), through researcher-driven engagement (Stage 2), and through re-contextualizing within a theoretical framework (Stage 3). The pictures were analyzed and discussed by all authors and integrated in the text analysis. Pictures that illuminated the themes from the text analyses were chosen as illustrations.

At the beginning of each focus group, participants were informed about the purpose of the study and the limits of confidentiality and asked to sign a consent form. For the pupils, parents also signed a consent form (in advance). All participants were informed of the importance of securing consent from all persons that were photographed before pictures were taken. Gaining this consent was emphasized strongly with the pupils. The school already had a written agreement in place with all parents to use pictures of pupils for purposes such as our study. To ensure the anonymity of participants, they are not described by gender or age, only by the group they belonged to. The study was approved by the Norwegian Center for Research Data (55841/3/STM).

## 3. Results

**Figure 1:**
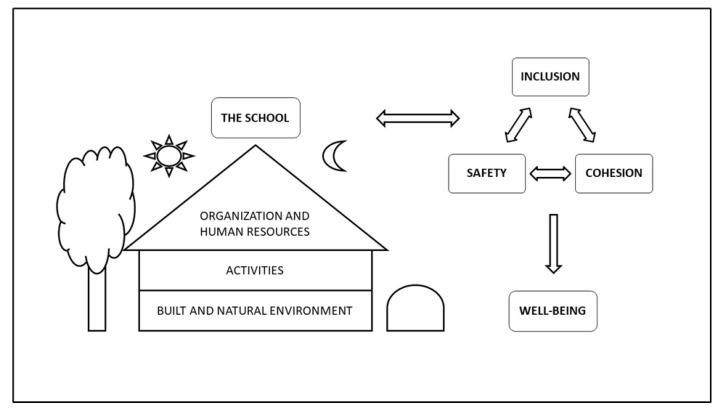
Factors associated with well-being in the community school.

The analysis of the text data and pictures revealed that four major themes were important for well-being. The first theme identified was the physical and organizational features of *the school*, such as the building and its surroundings, the activities happening there, and the human resources and organization. *The school* was the basis of the next three themes that emerged from the analyses, namely the perceptions of *safety, inclusion,* and *cohesion*. All these themes were interconnected, and they mutually influenced and enhanced each other and contributed to well-being (Figure 1).

### 3.1. The School

**Figure 2:**
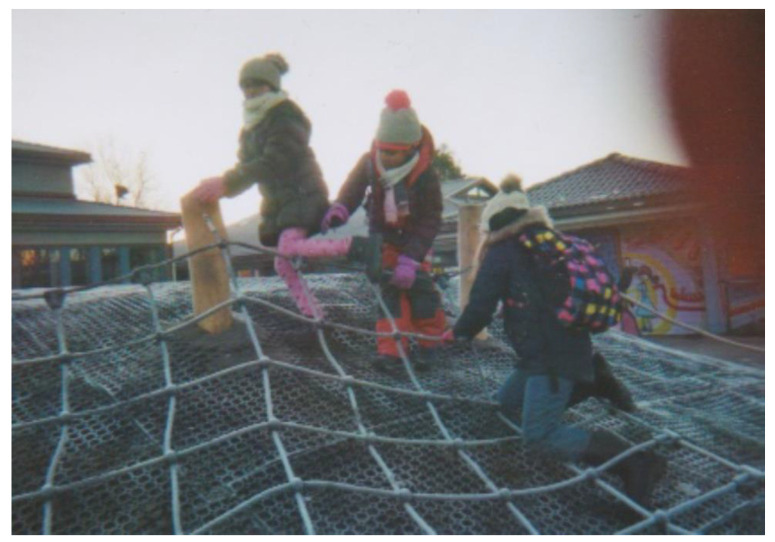
The Heap (photograph taken by Pupil 5).

The physical school building and its surroundings were important for all participants as a factor that encouraged well-being. The school library, kitchen and dining room, gym, venue for the leisure club “The mirror of the world”, playground, football field, graffiti paintings made by a local artist, and the large trees in the school yard were all objects in the pictures participants took to represent well-being. One special feature mentioned by all participants was the Heap (Figure 2). The Heap is a play structure in the school yard built together by pupils, parents, and school employees. For the pupils, the Heap was a source of well-being, as they could play there with their friends. One of them stated when discussing one of the pictures she had taken:

*I took this picture of these three children playing on our new heap. They seem to be enjoying themselves, and I like to play there myself (…) It’s a lot of fun, you can climb, and in the wintertime, you can slide down from it.*

(Pupil 5)

For the adult participants, the Heap was spoken of in terms of a symbol of the social engagement and level of co-creation between school employees and parents. The school contributed money for the materials, but most of the digging and building was done by parents and neighbors in their spare time:

*The school had some money put aside in order to fix the old Heap there, it was just a wearied and muddy Heap that could not be used for anything (…) As we could do a lot as voluntary work, it worked very well, and the costs were lowered (…) The principal, or team leader, prepared dinner for us (once) because we all came straight from work.*

(Parent 1)

As outlined, the school premises were used for a multitude of activities. All participants appreciated the variety of free activities, as it confirmed the school as an area for inclusion and equity. This was particularly true for the pupils and made it possible for them to experience the mastering of tasks and activities outside the curriculum, still within the school setting. One example that was photographed as a source of well-being by all stakeholders was the leisure club “The mirror of the world”, organized twice a week for the pupils by school employees and parents. One of the pupils spoke of their enjoyment of the leisure club:

*‘The mirror of the world” is very fun, because we have lotteries, we can go to the gym, and we can do handicrafts and make things, and it is very nice to be there (…) and there are lots of other children to play with and you never have to feel lonely, and it is so much fun to be there (…) and we meet other teachers than the usual ones and we never get as good meals as we do there.*

(Pupil 6)

**Figure 3:**
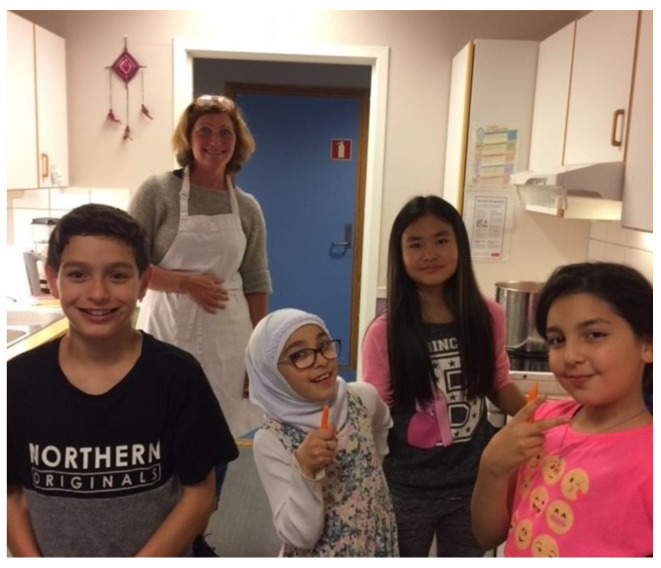
At the school kitchen (photograph taken by School employee 2).

The free school breakfasts were another activity highlighted by all participants as important for well-being. It was described as a forum where pupils, parents, school employees, and the principal could meet informally at the start of the day, providing an opportunity for all participants to have a nice start to their day. In addition to the social activity of meeting others over a meal, the preparation of meals both for breakfast and after-schooltime snacks was emphasized as an important social activity that promoted well-being (Figure 3):

*I try to attend the school breakfast as often as possible. It’s such a nice opportunity to talk to the pupils, to get to know them in a different way and learn what interests them … this morning the conversation was all FIFA gaming (laughing). As I am mostly stuck in my office during the day, I appreciate this opportunity to be close with the pupils, and sometimes the parents if they are joining, and when I see that the children are happy, it makes me feel happy… it creates well-being!*

(School employee 4)

The school employees expressed that working at a school where they were involved in activities both before and after regular working hours was a unique opportunity to get to know the pupils and their parents in a more profound way. Even though it required a lot of engagement and effort compared to working in a regular school, these additional activities were mainly seen as a pedagogical asset by the teachers. If any incident between the pupils had happened the evening before, they knew about it and could start the school day by addressing the issue:

*It’s not exactly a nine to five job, not an eight to six job either (…) But if the pupils experience well-being during the school day, then it will influence their leisure time. And if they experience well-being at the school during leisure time, they will bring that feeling into the classroom the next day.*

(School employee 3)

The human resources and organization at the school were also important elements that were emphasized by the participants. In particular, the parents referred to the human resources at the school as important for well-being:

*I believe the people working at the school are the most important resource. I mean you can have all the money in the world, you could have the most impressive building, or you could have a cardboard box. At the end of the day, it is the people in the organization or in the house who create the feeling of safety and inclusion … the belongingness.*

(Parent 4)

The commitment of earlier and current principals to physically open the school as a community arena was highlighted as an important premise for its successes.

### 3.2. Safety

**Figure 4:**
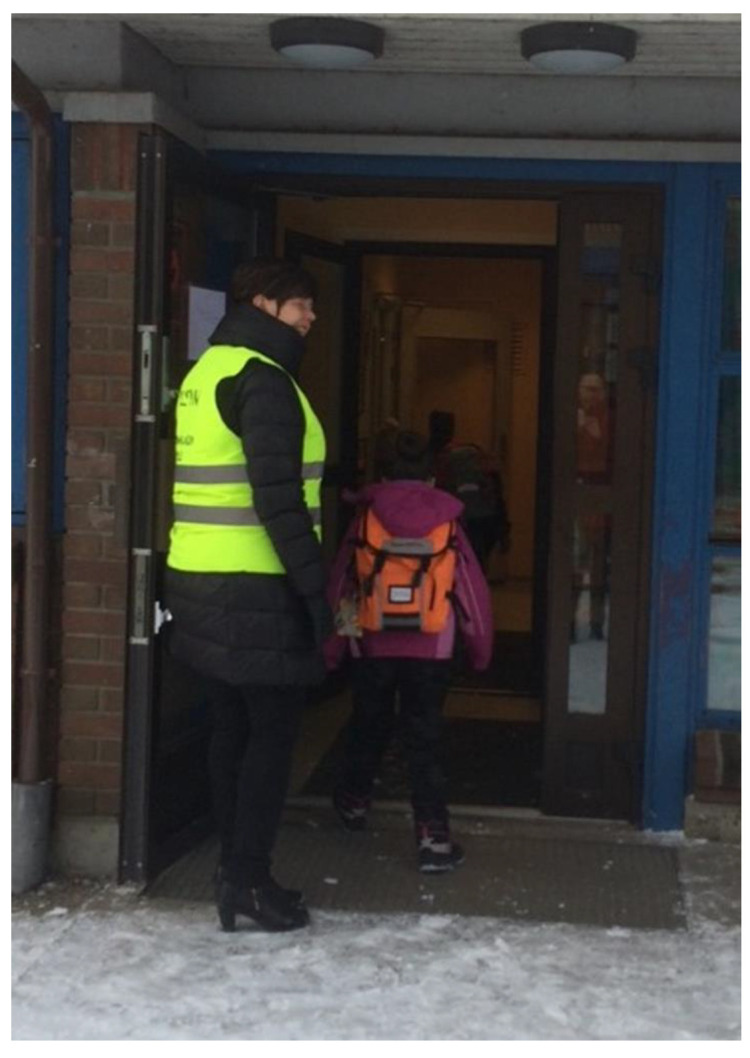
Principal greeting pupils every morning (photograph taken by Parent 2).

All the focus groups stated safety as a theme to ensure well-being at the school, and this was also a theme that emerged from the overall analysis. The parents stated that the lack of staff turnover created a safe environment for their children and that this contributed to both children and parent’s well-being. In addition, the school management’s philosophy of emphasizing a safe and inclusive environment also contributed to well-being. A tangible example of this philosophy put into action that was highlighted by parents was that each morning every single pupil was greeted by their name and welcomed to school by the principal (Figure 4).

The pupils also discussed how perceived safety was important for their well-being, represented in the pictures they took of the school traffic patrol, the friendship bench, and the peer well-being ambassador, illustrating how they felt safe walking to and from school, and ensuring that they would be included in social play during break time. One of the pupils referred to safety when explaining why her picture represented well-being for her:

*And on this picture, you can see the school traffic patrol, and they stop the traffic so that the small pupils can cross the road safely … and you feel safe … because even if the cars drive fast, the patrol ensures that they slow down (…) I think the school patrol creates well-being because it make us feel safe.*

(Pupil 1)

The school employees discussed the organizational and work environment that gave them room to experiment and where they felt safe to share their opinions. They also felt that the school’s long-term approach of inclusion and equity had built a safe environment for the pupils and the parents:

*Already many, many years ago I decided that those who walk into this school … inside this school, everybody should experience well-being (here). No matter how your life is on the outside (…) being in here it should feel good. In this school it will be acceptance, you will be accepted. You shall feel safe!*

(School employee, 6)

### 3.3. Inclusion

All participants expressed that an important feature that made them experience well-being at the school was a feeling of inclusion. The pupils expressed that being with friends and having fun was important for well-being, and the organized leisure time activities and the open playgrounds and football fields on the school premises made it possible to always find other children to play with. To encourage a sense of inclusion early in a child’s life, families with small children from the community were also involved in activities at the school. For example, in Norway, four year olds have an obligatory health check, and these were held on the school premises; this gave school employees an opportunity to meet families and get to know them before the child reached school age. The school employees felt that this was especially useful for immigrant families, as they could familiarize the families with the school and the community activities there at an early stage. Furthermore, it gave an opportunity to be in contact with families that needed extra resources or support:

*Today we had the four-year control, and the children come with their parents, so we get a chance to talk to the parents, to connect with them (…) In this school we meet children from so many cultures and with different “baggage”… after all, we are situated in this neighborhood which has its challenges… so we meet a lot of children that we know we really mean a lot for… we often get to know that we made a difference, right? And to know that you really made a difference, that’s just so valuable.*

(School employee 6)

Several of the activities that took place after school were mentioned by the participants as important for inclusion. The physical activity group for women made participation and inclusion possible for local women who otherwise, due to their religious or cultural backgrounds, would not participate in physical activities. Moreover, as the school was regarded as a safe place, many immigrant families felt secure letting their children participate in after school activities. Another example was the possibility to use the school premises for arranging birthday parties:

*Usually, the whole class is included and the classes are big … many families live in small flats in our community so it’s easier to gather at school, and you can use the sport facilities, and other facilities and the kitchen if you want to cook something. So, it’s really practical (…) It’s kind of ensuring equity really, because everybody gets the possibility to have a party, or at least given the opportunity to make it happen.*

(Parent 3)

The multitude of free activities after school and the free outlet of equipment for winter sport activities made it possible to include all children in activities regardless of the families’ economic situation. At the leisure club and the international café that was regularly arranged, there was a focus on including and representing the multicultural nature of the community. This was reflected in the pictures taken by all three groups of participants. The international café was also popular among the pupils’ extended families and other members of the community. These activities were important arenas for inclusion, and both children and parents took pride in being able to share aspects of their culture, such as music, national costumes, or food:

*And at the café everybody brings food, and there are dishes from all around the world, and the children are so proud to present candy from Poland, or cakes from Norway or wherever … and when sitting around the table with the other families you realize that we are all the same, we are all part of this community.*

(Parent 4)

The school employees also stated that the multicultural nature of the wider community was rewarding.

### 3.4. Cohesion

Both the school employees and the parents talked about a strong sense of cohesion as one of the factors that generated well-being at the school. The school employees had all worked at the school for several years and expressed pride and ownership for their roles in developing and cultivating the school as a community arena. This had developed into a strong cohesion at the school and also in the community:

*We (the school) want to be a place where everybody can meet. Where everyone is equally important. We strive to create a ‘we-culture’ at the school and in the community.*

(School employee 3)

The pupils did not explicitly express this sense of cohesion; however, both their pictures and their discussion revealed they felt the school was a safe and comfortable environment where they felt ‘at home’ and where they could meet friends:

*I think the reason why we so much like being here, is that we have classes, but then we have things to do after classes at the school in the evening, and that’s why I like it so much here, because we always have all our friends right here.*

(Pupil, 1)

This sense of cohesion was also shared by the parents, as one said:

*On the soccer field parents are often playing with the kids, and I think it creates a great sense of cohesion because the children get to know each other’s parents. It’s a small community and the children know us, like when you approach the school someone will shout: “there comes your mum to pick you up!”. So, it’s a very nice gathering place. If there is nobody home to play with in your street, you could always ride your bike to the school to find somebody to play football with, so it’s definitely the gathering place for the community.*

(Parent 1)

**Figure 5:**
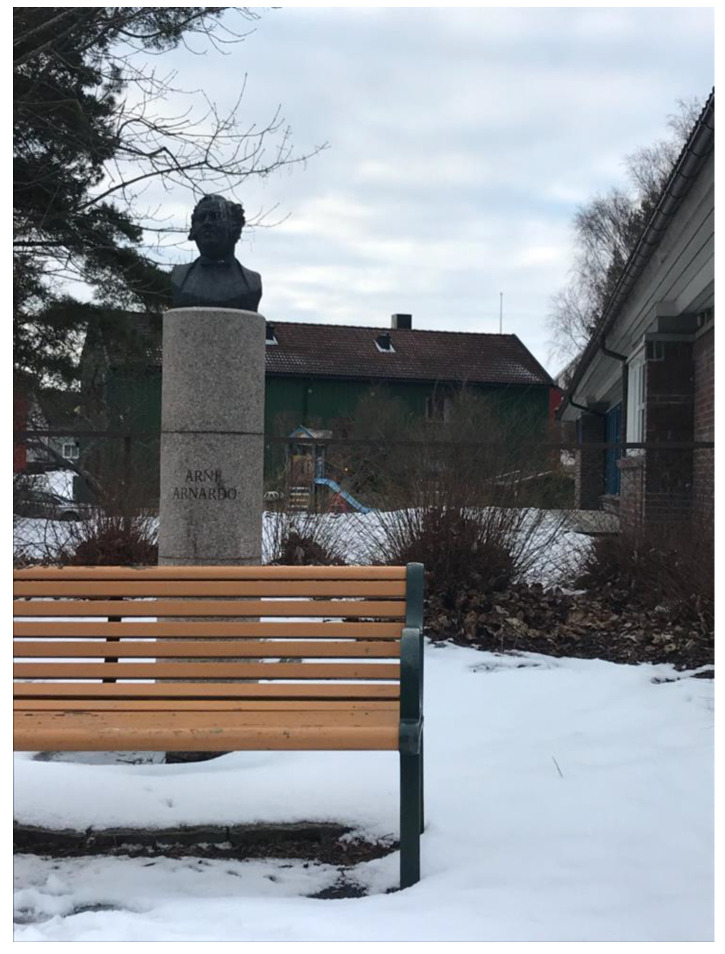
Cohesion (photograph taken by Parent 4).

All the participants referred to the school and their community with a sense of pride despite the many challenges in the neighborhood. The school has received several awards for their work as a community arena, and this enhanced the pride felt by the community of what the school had achieved. One of the parents had taken a picture of a statue outside the school yard (Figure 5) and explained why she thought this illustrated what the school meant for cohesion and well-being in the community:

*And this is a picture of the statue of Arnardo right outside the school. That’s kind of illustrative of this community. He came from here, from nowhere, and then became the king of Circus in Norway, and we are so proud of him. And there you see the bench where old people sit and chat. And there is the playground of the old kindergarten, and then there is the school (…) and the bushes by the statue are super exciting for the small children to play in … so all of this in a way represents how important the school is in this community, connecting everybody and making us feel that we belong together. After all, the node of our community is the school, it’s like a little nerve center.*

(Parent 4)

## 4. Discussion

The main findings of this study show that the features of the school, such as the built and natural environment, the activities happening there before and after school time, and the human resources and organization at the school, all facilitated perceptions of safety, inclusion, and cohesion among pupils, school employees, and parents. Together, these elements led to well-being in all participants and facilitated participation and equity in the community.

Through both pictures and focus group discussions, all participants stated that the school’s built and natural environment were important elements for engaging in non-education activities and contributed to a feeling of cohesion associated with well-being. During the last decade, there has been an increased focus on how place, e.g., the built and natural environment, can contribute to well-being [41,42,43]. Qualities of the built environment, such as design, aesthetic quality, and accessibility, have been found to be associated with social cohesion and social capital [44,45]. Elements of the built environment, such as the socker field and the playground, enhanced community interactions and participation and created a shared identity. Places such as this, that create shared identities, have also been found to be associated with increased neighborhood networking, higher social cohesion, and enhanced place attachment [46].

One of the important pillars of community schools, especially in deprived areas, are the activities that take place before and after the typical school day [47]. These activities should complement, rather than duplicate, the regular school day and provide pupils with opportunities to pursue their own interests and to develop socially, emotionally, and physically [47]. In our study, both the multitude of free activities offered at the school before and after school time and the parents’ perception of the school as a safe place for their children to be facilitated pupils to be able to engage in activities regardless of their socioeconomic or ethnic background. For the pupils, the range of activities offered after school gave them opportunities to meet friends and improve the mastering of tasks unrelated to the curriculum, although still experienced as mastering in the school setting. This created positive feelings toward the school. Research on school-based extracurricular activities has been linked to many positive outcomes, such as better psychological adjustment, higher self-esteem, reduced feelings of social isolation, and reduced rates of antisocial behavior [48]. Although not within the scope of our study, reduced rates of antisocial behavior have been indicated by housing association leaders in the same neighborhood after the school was established as a community arena [33].

The pictures and text analysis showed the organization and human resources at the school as important factors for creating well-being for all participants. For a community school to succeed, a prerequisite is that the school organization must be open to cooperation, or even co-creation, with other stakeholders in the community [23]. The school staff and parents both highlighted the current and previous principals’ important role in initiating and developing the school’s premises, activities, and community engagement. This is in line with earlier research on community schools that have documented the principal’s key role for developing reciprocal community involvement [15,49]. The analysis also showed that equally important for the success of the community school was how the school staff defined their own professional identities and how they experienced the fluctuating borders between their working hours and their leisure time. Although the school staff described that extra efforts were needed, they still found their job meaningful and rewarding, and interacting with the pupils and their parents outside the classroom was regarded as a resource in their pedagogical work during school day. High staff well-being has also been reported in community schools when it is characterized by a sense of belonging, common goals, and meaningful and influential engagement [50].

An important aspect of the community school in this study is that it had developed gradually as a growing cooperation between school management and staff, pupils, parents, and the community [33]. It was also made very clear in the focus group meetings that the development of the school as a community arena was the result of a series of initiatives that were initiated from the bottom-up, through processes that clearly fit within the framework of co-creation. An illustrative example of co-creation at the school was the creation of the popular physical structure known by the community as the Heap. When building the Heap, different actors contributed with different resources; the school contributed with organizing and funding, the parents contributed with work hours, and a local entrepreneur contributed with the know-how and machinery. In addition to creating a publicly available common good in the community, the process of co-creation of the Heap in itself was reported as strengthening ties between the stakeholders, as the work facilitated participation and became a social happening. Moreover, the international café, which was co-created by the school staff, pupils, parents, and other community members, was an arena for participation, integration, and inclusion. Here, all community members from different socioeconomical and ethnic backgrounds could participate on equal terms, contributing with food or doing other tasks. Both the Heap and the international café were activities that led to increased participation and thereby built cohesion and trust in the community. Building trust amongst different actors has been shown to be a key factor in generating social capital and thereby succeeding in co-creation [22]. The participants all emphasized the fact that they had been building long-term relationships with each other and that these relationships were the foundation for the current feelings of safety, inclusion, and cohesion.

This study has limitations and strengths that need to be considered. We used photovoice as a method, which is originally developed as a participatory action research approach that involves mobilizing information and action back to the local community [34]. However, the scope of our study did not allow for actively doing this, although all pictures and results were made available to the school for use in future exhibitions, meetings, or other community activities. The methodology still provided a participatory approach that ensured the different stakeholders had the active part in deciding what topics and themes were important for them to create well-being. Moreover, we used photovoice to target and focus upon positive elements of the school, which might enhance positive feelings and raise awareness and pride of the positive qualities of the school [51]. This is a strength, as this method is more often used to explore negative factors [52]. Another limitation of the study was the use of a convenience sample, as this might increase the risk of sampling bias. We have no information on the representability of the sample, and bias cannot be ruled out. It could be argued that willingness to participate might be associated with more positive attitudes toward the school. However, the aim of this study was not to capture all possible experiences of the school but to investigate which elements created well-being for the participants. Still, there are reasons to expect that the convenience sample could be biased toward more resourceful individuals, as the school’s pupil’s and parent’s council were used to recruit participants. The number of participants was relatively small but within the number often reported for photovoice studies [36]. Moreover, when the information power of the data is high and relevant for the study, as in this study, a lower number of participants is needed [53]. To strengthen the credibility of the findings, all authors analyzed the data, and none had any connections to the school or the community.

## 5. Conclusions

This study showed that the school’s built and natural environment, the activities happening there, and the human resources and organization at the school all facilitated perceptions of safety, inclusion, and cohesion. Developing a school as a community arena could therefore be an innovative way of building participation, equity, and well-being in communities. Our findings could also be of relevance for developing schools as community arenas outside Norway or Scandinavia, and such an approach might be especially important in deprived areas or in communities with inhabitants from diverse ethnic backgrounds. Schools that want to develop as a community arena should open their premises for diverse use and activities initiated by pupils, parents, and other community members. This requires that the school’s staff is open to engage in co-creation with other stakeholders in the community. 

## Data Availability

Data supporting reported results are kept at The Norwegian University of Life Sciences.
